# *daf-2* modulates regeneration of mechanosensory neurons I

**DOI:** 10.17912/W2XD3R

**Published:** 2017-12-01

**Authors:** Zehra C. Abay, Michelle Yu-Ying Wong, Brent Neumann

**Affiliations:** 1 Neuroscience Program, Monash Biomedicine Discovery Institute and Department of Anatomy and Developmental Biology, Monash University, Melbourne VIC 3800, Australia

**Figure 1.**
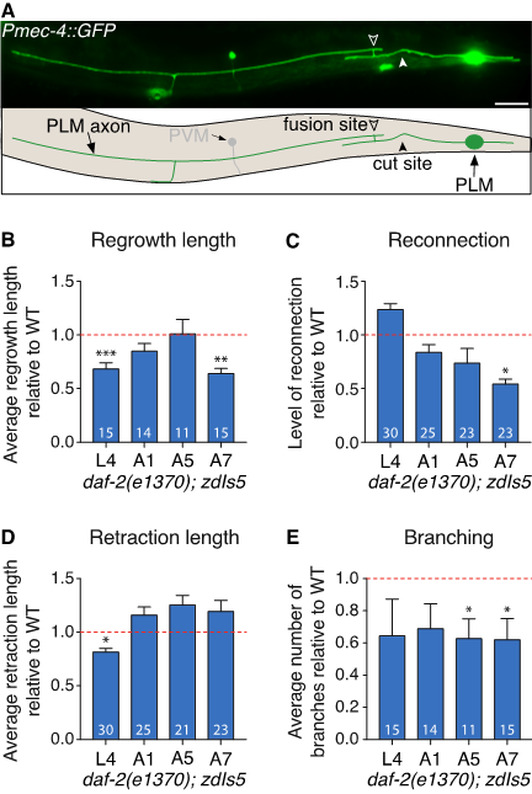
**The role of *daf-2* in axonal regeneration.** (**A**) Image and schematic of successful axonal fusion in a posterior lateral microtubule (PLM) neuron 24 h post-axotomy. Closed arrowhead shows cut site, open arrowhead shows fusion site. The posterior ventral microtubule (PVM) neuron is also visible in this image. Scale bar represents 25µm. (**B**) Quantification of the average length of regrowth, (**C**) level of reconnection, (**D**) average length of retraction, and (**E**) average number of branches in *daf-2(e1370); zdIs5* animals relative to wild-type (WT, *zdIs5*) across different ages. Dashed line designates a value of 1 (no change compared to WT). L4 = larval stage 4; A1 = one-day-old adult. *P* values from *t* test: * < 0.05, ** < 0.01, *** < 0.001; n values within each bar.

## Description

*Caenorhabditis elegans* (*C. elegans*) possess the ability to spontaneously regenerate injured axons via a highly efficient mechanism known as axonal fusion (Ghosh-Roy, et al., 2010; Neumann et al., 2011; Neumann et al., 2015; Abay et al., 2017). Following laser axotomy, regrowth from the proximal axon segment (still attached to the cell body) reconnects and fuses with its separated distal segment (Fig. 1A). We recently demonstrated that the level of axonal fusion increases with age (Abay et al., 2017). The *daf-2* gene encodes an insulin-like growth factor/IGF-1 receptor that has previously been shown to inhibits neurite regeneration in an age-dependent fashion (Bryne et al., 2014; Kravtsov et al., 2017). To determine if DAF-2 functions in a similar fashion in the mechanosensory neurons to mediate the age-dependent modulation of axonal regrowth and axonal fusion, we studied axonal regeneration in the posterior lateral microtubule (PLM) neurons of animals carrying the *daf-2(e1370)* mutation.

*daf-2* mutants displayed significantly reduced regrowth of PLM specifically at the final larval stage (L4), and in seven-day-old adults (A7) (Fig. 1B). Mutation of *daf-2* also reduced the level of reconnection in A7 animals, but had no effect at other ages (Fig. 1C). The level of successful axonal fusion was not affected by the *daf-2* mutation at any age. As *daf-2* also mediates age-dependent changes in retraction length after transection of motor neurons (Byrne et al., 2014), we next quantified the length of retraction between the severed ends of the PLM axon. The length of retraction significantly decreased in L4 stage *daf-2* mutants, but was unchanged in adult stages (Fig. 1D). The average number of branches was reduced in *daf-2(e1370)* animals across all ages analysed, with significant reductions observed in A5 and A7 animals (Fig. 1E). ​

Overall, our results imply that mutation of *daf-2* does lead to a linear relationship between lifespan extension and modulation of regeneration in the PLM mechanosensory neurons.

## Reagents

Hermaphrodites were used for all experiments, and were grown under standard conditions at 20°C. The QH4370 [*daf-2(e1370); zdIs5(Pmec-4::GFP)*] strain was used along with the QH3135 [*zdIs5(Pmec-4::GFP)*] control strain. The *daf-2(e1370)* allele has been considered temperature sensitive for the dauer phenotype, but not for the long-lived phenotype. At 20°C, *daf-2(e1370)* animals display a greater than 2-fold increase in lifespan compared to the wild-type (Kenyon et al., 1993). Laser axotomy, microscopy and quantification of data was performed as previously described (Abay et al., 2017).
